# Add‐On Telitacicept Significantly Improves Outcome of Patients With Refractory Ocular Myasthenia Gravis a Real‐World Case Series

**DOI:** 10.1002/brb3.71157

**Published:** 2025-12-31

**Authors:** Jing Lin, Yue Li, Mengcui Gui, Bitao Bu, Zhijun Li

**Affiliations:** ^1^ Department of Neurology, Tongji Hospital, Tongji Medical College Huazhong University of Science and Technology Wuhan Hubei China

**Keywords:** B‐cell activating factor, minimal symptom expression, refractory ocular myasthenia gravis, telitacicept

## Abstract

**Introduction:**

Refractory ocular myasthenia gravis (MG) represents a significant therapeutic challenge, as conventional immunotherapies often prove ineffective. Telitacicept, a recombinant B‐lymphocyte stimulator receptor‐antibody fusion protein, offers a novel immunomodulatory approach. This retrospective study evaluates its efficacy and safety in patients with MG‐associated refractory ocular symptoms.

**Methods:**

This is a single‐center retrospective cohort study. evaluated patients with refractory ocular symptoms who received telitacicept weekly between August 2024 and January 2025. We evaluated treatment efficacy using Myasthenia Gravis Foundation of America post‐intervention status (MGFA‐PIS), Myasthenia Gravis Impairment Index‐patient‐reported outcome (MGII‐PRO), quantitative MG (QMG), and MG activity of daily living (ADL) scores, as well as the reduction in daily prednisone dosage at baseline and each month following treatment. The safety assessment was also evaluated.

**Results:**

Seven MGFA class 1 patients (5 females, 2 males) were enrolled, with a median onset age of 6 years (interquartile range [IQR] 2–21) and a median disease duration of 98 months (IQR 46–121). MG‐ADL scores showed significant reduction with prolonged follow‐up. 6/7 patients achieved ≥2‐point MG‐ADL improvement by the third follow‐up. 4/6 patients reached minimal symptom expression at the fifth follow‐up. The average ocular MG‐ADL, MGII‐PRO, and QMG scores revealed statistically significant improvements at the third month of follow‐up, sustained through the sixth month. The regimen exhibited excellent tolerability, with no severe adverse events (e.g., hypersensitivity reactions, infections) reported.

**Conclusion:**

Our preliminary findings indicate that telitacicept represents a promising and well‐tolerated adjunct therapy for refractory ocular symptoms in MG, providing significant clinical evidence to support this novel therapeutic approach.

## Graphical abstract

6/7 patients achieved CMI by the third follow‐up, sustained to the fourth follow‐up, and 4/6 reached MSE by the fifth follow‐up.

First real‐world evidence of adding telitacicept efficacy in refractory ocular MG symptoms

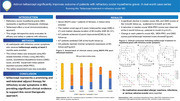

**Abbreviations**
AChEIs: acetylcholinesterase inhibitors
AChR: acetylcholine receptor
AZA: azathioprine
CMI: clinical meaningful improvement
GC: glucocorticoid
gMG: generalized MG
IQR: interquartile range
IVIG: intravenous immune globulin
MG: myasthenia gravis
MG‐ADL: MG‐related Activities of Daily Living
MGFA‐PIS: Myasthenia Gravis Foundation of America post‐intervention status
MGII‐PRO: Myasthenia Gravis Impairment Index‐patient‐reported outcome
MMF: mycophenolate mofetil
MSE: minimal symptom expression
OMG: ocular MG
QMG: Quantitative Myasthenia Gravis
TAC: tacrolimus

## 1 Introduction

Ocular myasthenia gravis (OMG), a distinct subset of myasthenia gravis (MG), is an autoimmune disorder affecting the neuromuscular junction, mediated through autoantibodies that target postsynaptic membrane proteins, primarily the acetylcholine receptor (AChR) and muscle‐specific kinase (Dresser et al. 2021). OMG is characterized by fluctuating muscle weakness and pathological fatigability, with clinical manifestations strictly limited to the ocular musculature in approximately 15% of MG patients (Gilhus NE. 2016). Approximately 60%–80% of patients initially present with extraocular muscle weakness, which partially progresses to generalized MG (gMG) within 2 years (Guo et al. 2022). The median duration of transformation and conversion rates were also inconsistent in patients with generalized onset with varying ages of onset, such as childhood‐onset and adult‐onset, as reported (Bi et al. 2023; Bi et al. 2022). Furthermore, patients with partial gMG may experience refractory residual ocular symptoms after standard treatment with immunosuppressants (Tomschik et al. 2020). Notably, early immune intervention is particularly necessary for patients at high risk of transformation from OMG to gMG (Bi et al. 2022; Ma et al. 2024).

Symptomatic treatments, such as acetylcholinesterase inhibitors (AChEIs), are commonly prescribed to patients with OMG. Corticosteroids are recommended for patients demonstrating an inadequate response to AChEIs (Narayanaswami et al. 2021). Immunosuppressants or thymectomies may benefit patients unresponsive or intolerant to corticosteroids (Bi et al. 2022; Narayanaswami et al. 2021; Zhang et al. 2022).

Refractory OMG is clinically defined as cases that do not respond to multiple conventional treatments, experience intolerable adverse drug reactions, or suffer frequent disease relapses, making it difficult to achieve treatment goals, which is similar to the refractory cases in gMG (Haghikia et al. 2023; Howard et al. 2017). However, there is currently no reported relative incidence of refractory OMG. Refractory ocular symptoms, including refractory OMG and residual ocular symptoms in gMG, significantly impair daily functioning and cause considerable psychological distress, presenting a pressing clinical challenge.

Biologically targeted therapies such as neonatal Fc receptor antagonists and complement C5 inhibitors represent new treatment strategies for patients (Howard et al. 2017; Zhong et al. 2024; Howard et al. 2021; Bril et al. 2023; Howard et al. 2023; Antozzi et al. 2025). The effectiveness of some biological agents has also been reported in certain cases involving OMG or gMG patients with residual ocular muscle symptoms (Ma et al. 2024; Weidmayer and Gallagher 2023; He et al. 2025). Telitacicept, a recombinant fusion protein comprising a transmembrane activator and calcium‐modulating cyclophilin ligand interactor, exhibits a significant role in blocking both B lymphocyte proliferation and T lymphocyte maturation (Dhillon 2021). Approval in China for systemic lupus erythematosus (SLE) in March 2021, telitacicept showed promising therapeutic efficacy and safety in gMG in a phase II clinical trial (Yin et al. 2024). Telitacicept was approved in China for gMG in May 2025. Given prior evidence in autoimmune diseases, including MG, further clinical investigations are needed to optimize therapeutic strategies for these refractory ocular symptoms.

This retrospective study evaluated the therapeutic potential of adding telitacicept in MG patients with refractory ocular symptoms.

## 2 Participants and Methods

### 2.1 Study Design and Participants

This single‐center retrospective cohort study was conducted between August 2024 and January 2025 to investigate add‐on telitacicept therapy in patients with refractory ocular symptoms at Tongji Hospital of Tongji Medical College, Huazhong University of Science and Technology. All patients were definitively diagnosed with Class I MG according to the Myasthenia Gravis Foundation of America (MGFA) classification system prior to telitacicept initiation and had received telitacicept for at least three months. The diagnosis of MG was established based on the following criteria: (1) typical clinical manifestations featuring characteristic fluctuating muscle weakness and fatigability; (2) objectively positive responses in the neostigmine test; (3) abnormal neuromuscular transmission as confirmed by repetitive nerve stimulation; and (4) the presence or absence of positive anti‐AChR‐Abs. Differential diagnosis was conducted for child‐onset OMG, and the following conditions were excluded: (1) congenital myasthenic syndromes; (2) atypical Graves' disease; and (3) Kearns–Sayre syndrome.

This study was approved by the Clinical Investigation Committee of Tongji Hospital, Tongji Medical College, Huazhong University of Science and Technology (approval number. TJ‐IRB202406006) in full compliance with the Declaration of Helsinki. Patients were conducted economic assessment and signed an informed consent form for clinical evaluation and follow‐up before enrollment in the study.

### 2.2 Efficacy Assessment

All patients received subcutaneous telitacicept injections at a dosage of 80–160 mg weekly, depending on their body weight. The maintenance duration of the treatment and follow‐up period was no less than 3 months. Concomitant immunosuppressive therapies, comprising steroids and immunosuppressants, were continued as part of the treatment regimen.

The patients were evaluated before and after telitacicept treatment. The “baseline” was defined as the last assessment conducted before the initial telitacicept dose. The demographic characteristics and clinical manifestations of each patient were documented, including the age of onset, disease duration, MGFA class, antibodies, comorbidities, daily dose of prednisone (or equivalent dose of methylprednisone), and types of immunosuppressive agents. MG‐related Activities of Daily Living (MG‐ADL), Quantitative Myasthenia Gravis (QMG), and Myasthenia Gravis Impairment Index (MGII) (Jaretzki et al. 2000) scores were used to evaluate treatment efficacy at each visit. The ocular subscale of the MGII scale includes patient‐reported outcome (PRO) and physician‐examined (PE) items. PRO items were selected as the primary evaluation metric, as QMG ocular scores partially overlap with PE items. Minimal symptom expression (MSE), a new concept introduced in MG research over the past two years, is defined as an MG‐ADL score of 1 or less. MSE shows significant potential for measuring the efficacy of MG therapy, especially in the context of emerging MG treatments (Muppidi et al. 2022). Clinically Meaningful Improvement (CMI) is defined as a reduction of at least 2 points in the MG‐ADL score compared to the baseline (Muppidi et al. 2011). Clinical assessments were performed at baseline and each month after treatment.

Additionally, standardized clinical measures, including MG‐ADL, MGII‐PRO, and QMG scores, and the reduction in daily prednisone dosage were evaluated to determine the efficacy of telitacicept. Moreover, QMG assessments were conducted 6–8 h after pyridostigmine administration to avoid the potential influence of anticholinesterases.

All adverse reactions during treatment, including noninfectious, infectious, and hypersensitivity reactions, were collected and graded according to the Common Terminology Criteria for Adverse Events.

### 2.3 Statistical Analysis

Statistical analyses were performed using SPSS 27.0 (IBM Corp., NY, USA), and graphical drafting was generated with GraphPad Prism 9.2.0 (GraphPad Software, San Diego, USA). Descriptive analysis was conducted to describe the demographic and clinical characteristics of the patients. Categorical variables and count data were presented as [n (%)]. Continuous variables were presented as mean ± standard deviation for normally distributed data or as median and interquartile range (IQR) for non‐normally distributed data. Two groups were compared using an independent sample t‐test or corrected t‐test, and the Mann‐Whitney U test was used for two‐group comparisons. For the differences in each index at different follow‐up times, normally distributed data were analyzed using a one‐way repeated‐measures analysis of variance, and non‐normally distributed data were analyzed using the Friedman test. The significance level was set at *p* = 0.05. *p* < 0.05 was considered to be statistically significant.

## 3 Results

### 3.1 Baseline Characteristics of Patients

The retrospective study comprised seven patients with refractory ocular symptoms. All patients exhibited MGFA class 1 expression at baseline before the addition of telitacicept. Of these, five patients (cases 1–5) consistently presented with OMG throughout the course of the disease, whereas the remaining two patients (cases 6 and 7) were diagnosed with gMG at the peak of the disease. The initial symptom in patients 6 and 7 was external ophthalmoplegia, which progressed to gMG after 120 and 280 months, respectively. Both patients experienced myasthenic crises during acute exacerbations. Following rapid rescue treatment (efgartigimod and intravenous immunoglobulin [IVIG]), the respiratory muscle and limb strength improved, but residual extraocular muscle weakness persisted.

**FIGURE 1:**
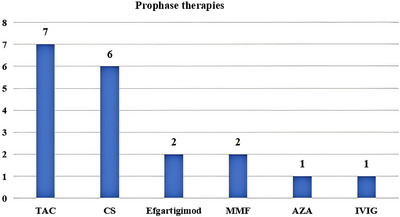
Types of immunosuppressive agents previously administered to patients with refractory ocular symptoms. CS, corticosteroids; TAC, tacrolimus; MMF, mycophenolate mofetil; IVIG, intravenous immune globulin; AZA, azathioprine.

At baseline, prior to the initiation of telitacicept treatment, the median age of the seven patients was 21 years (IQR 11–25), comprising five females and two males. The median age at disease onset was 6 years (IQR 2–21), with a median disease duration of 98 months (IQR 46–121). Among them, five had childhood‐onset disease (onset age <18 years), and two had early‐onset disease (18 < onset age ≤ 50 years) (Table 1). All patients received pyridostigmine as initial symptomatic treatment. In Case 4, pyridostigmine was discontinued due to intolerance to adverse effects, such as abdominal pain. Six patients were initially treated with corticosteroids as first‐line immunotherapy, with a median dosage of 18.33 mg and a median disease duration of 38 months (IQR 2–110) at initiation. Notably, Patient 2 declined steroid therapy due to concerns over potential adverse reactions. Patients 1, 4, and 5 discontinued corticosteroids because of side effects, including growth retardation, Cushing's syndrome, and diabetes mellitus. Patients 3 and 6 were maintained on steroids prior to the addition of telitacicept, with daily doses of 15 mg for patient 3 and 5 mg for patient 6. Before baseline, seven patients had received two or more types of immunotherapies, with a median of three (IQR 2, 3) (Figure 1). At baseline, patients 1, 2, 4, and 6 received tacrolimus (TAC) treatment simultaneously, whereas patients 3 and 5 received MMF treatment. The reasons for adding telitacicept in these seven patients were as follows: symptoms severely affecting daily studies and work (7/7), intolerance to treatment side effects (3/7), and poor efficacy of previous treatments (5/7).

**TABLE 1:** Baseline demographic and clinical characteristics of patients with refractory ocular symptoms in myasthenia gravis.

Patient No.	Sex	Onset age (Y)	Baseline age (Y)	Disease duration (M)	Onset symptoms	Autoantibody subtypes	Thymus disease	Comorbidities	MGFA class at the peak of disease	Prophase immunotherapies	Treatment at baseline (daily)	MGFA class at baseline
1	M	3	11	98	ptosis	AChR‐Ab	None	Thyroid disease	1	CS, TAC	Pyr 90 mg, TAC 2mg	1
2	F	21	21	7	Ptosis, diplopia	AChR‐Ab	None	Hyperthyroidism	1	TAC, Efgartigimod	Pyr 180 mg, TAC 3mg	1
3	F	15	23	97	ptosis	AChR‐Ab	Not in detail	/	1	CS, TAC, MMF	Pyr 180 mg, CS 15 mg, MMF 0.5 g bid,	1
4	F	28	32	46	ptosis	Negative	None	/	1	CS, TAC	TAC 3mg	1
5	M	2	13	105	ptosis	AChR‐Ab	None	Neurofibroma	1	CS, TAC, MMF	Pyr 180 mg, MMF 0.5 g bid	1
6	F	1	11	121	ptosis	Negative	Not in detail	/	5 (myasthenia crisis)	CS, TAC, AZA, Efgartigimod	Pyr 180 mg, CS 5 mg, TAC 2 mg,	1
7	F	6	25	230	ptosis	Negative	None	/	5 (myasthenia crisis)	CS, TAC, IVIG	Pyr 180mg	1
Patient No.	Duration of baseline symptoms (M)	Symptoms and signs at baseline	Reasons for the addition of telitacicept	Protocol of telitacicept	Maintain the duration of the telitacicept treatment							
1	8	Ptosis in both eyelids, fixed both eyeballs	unresponsive to standard therapies, side effects of therapies, quality of life significantly affected	80 mg once, weekly	5 months							
2	7	Ptosis in both eyelids, fixed both eyeballs	unresponsive to standard therapies, quality of life significantly affected	160 mg once, weekly	8 months							
3	12	Ptosis in both eyelids, fixed both eyeballs	unresponsive to standard therapies, quality of life significantly affected	160 mg once, weekly	9 months							
4	19	Ptosis in both eyelids, almost fixed both eyeballs with very slight horizontal movement	Side effects of CS: Cushing syndrome, intolerance of the side effects of Pri, quality of life significantly affected	160 mg once, weekly	6 months							
5	31	Diplopia, ptosis in left eyelid, limited abduction of the left eye and fixed eye movement in right eyeball	unresponsive to standard therapies, quality of life significantly affected	160 mg once, weekly	5 months							
6	8	Ptosis in both eyelids, fixed both eyeballs	Side effects of CS: Cushing syndrome and developmental retardation, quality of life significantly affected	80 mg once, weekly	6 months							
7	18	Diplopia, ptosis in left eyelid, limited abduction and upward gaze of the left eye	unresponsive to standard therapies, quality of life significantly affected	160 mg once, weekly	7 months							

Abbreviations: AchR‐Ab, anti‐acetylcholine receptor antibody; CKD, chronic kidney disease; CS, corticosteroids; DM, diabetes mellitus; F, female; HTN, hypertension; IVIG, intravenous immune globulin; M, male; M, months; MMF, mycophenolate mofetil; MuSK‐Ab, muscle‐specific tyrosine kinase; Pyr, pyridostigmine; TAC, tacrolimus; Y, years.

**FIGURE 2:**
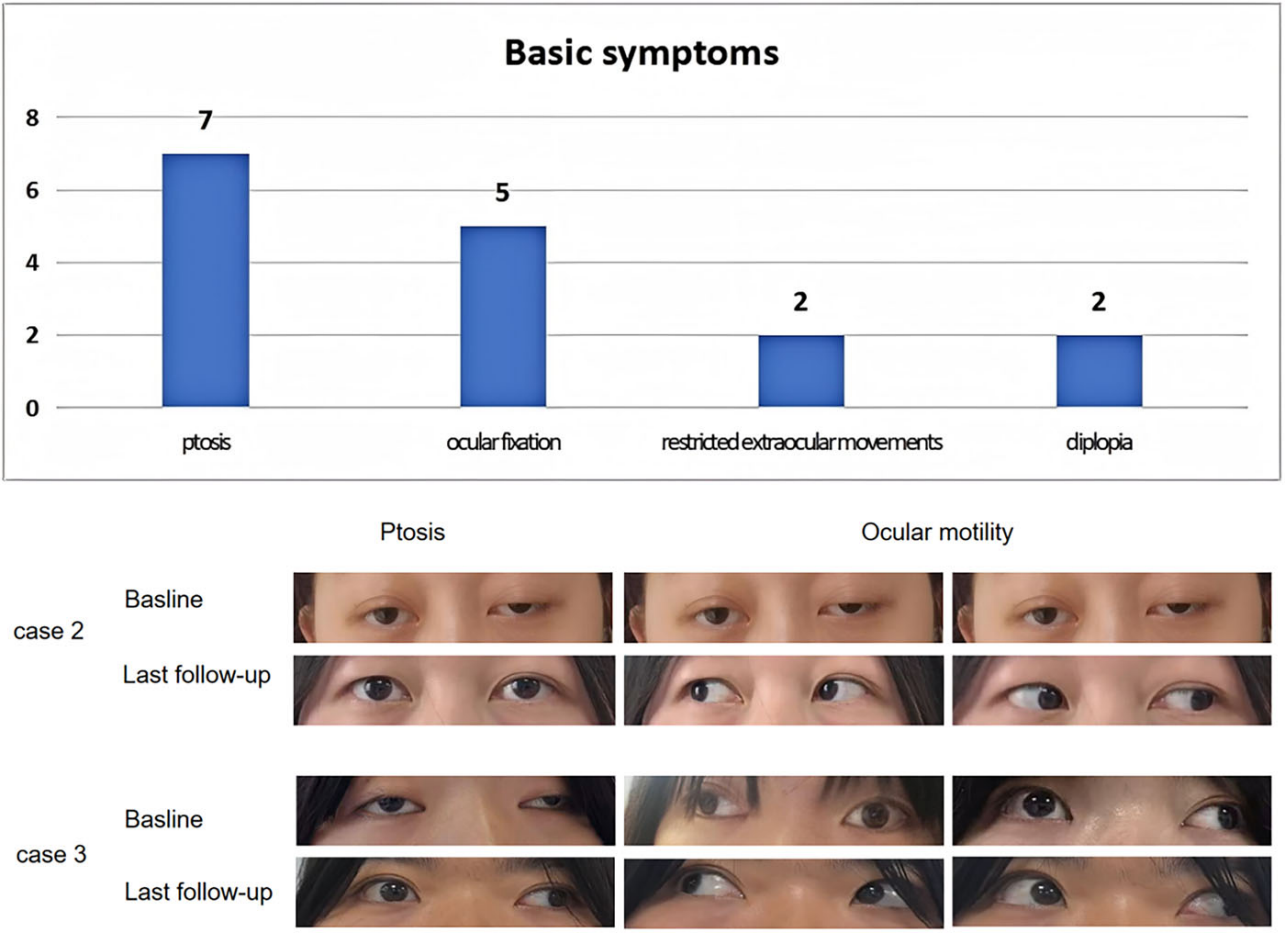
Ocular symptoms in this study: (A) Basic ocular symptoms observed in the seven patients at baseline. (B) Improvements in ptosis and ocular motility were observed in both Case 2 and Case 3 at baseline and at the final follow‐up.

The ocular symptoms observed in the seven patients at baseline mainly included ptosis (7/7), ocular fixation (5/7), restricted extraocular movement (2/7), and diplopia (2/7) (Figure 2). The median duration of baseline symptoms was 12 months (IQR 8, 19). At baseline, the average ocular ADL score was 5.74 ± 0.49, the ocular MGII‐PRO score was 14.29 ± 1.98, and the ocular QMG score was 6.00 ± 0.00. All patients received subcutaneous telitacicept once weekly as an add‐on therapy. Patients 1 and 6 were administered 80 mg weekly due to lower body weight, whereas the remaining patients received 160 mg weekly. The median maintenance duration of telitacicept therapy was 6 months (IQR 5, 8), and the median follow‐up period was 7 months (IQR 5, 8).

### 3.2 Effectiveness of Telitacicept Therapy

**FIGURE 3:**
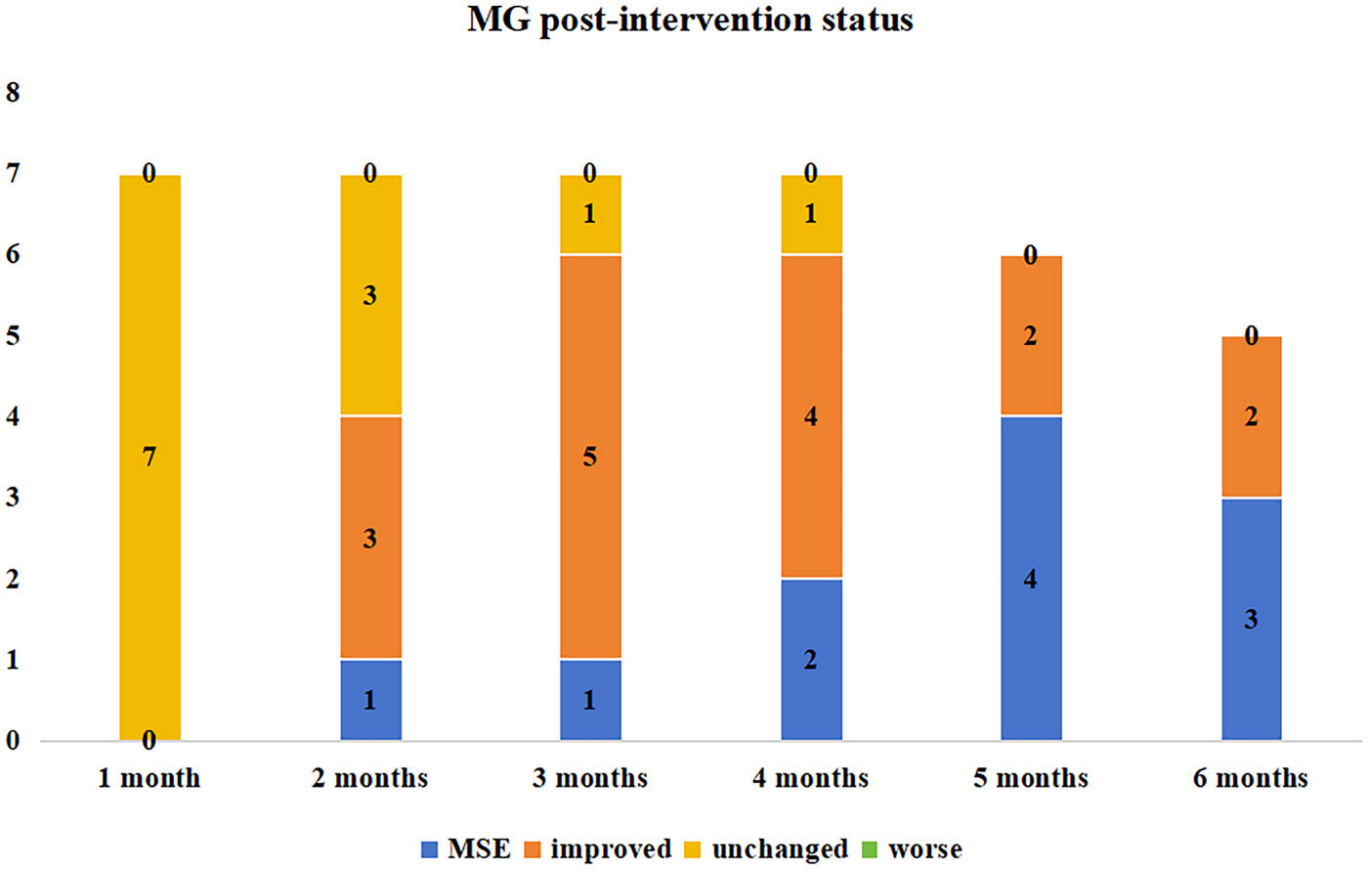
Assessment of clinical status using MGFA‐PIS post‐telitacicept treatment. MG‐PIS, Myasthenia Gravis Foundation of America post‐invention status; MSE, minimal symptom expression.

**FIGURE 4:**
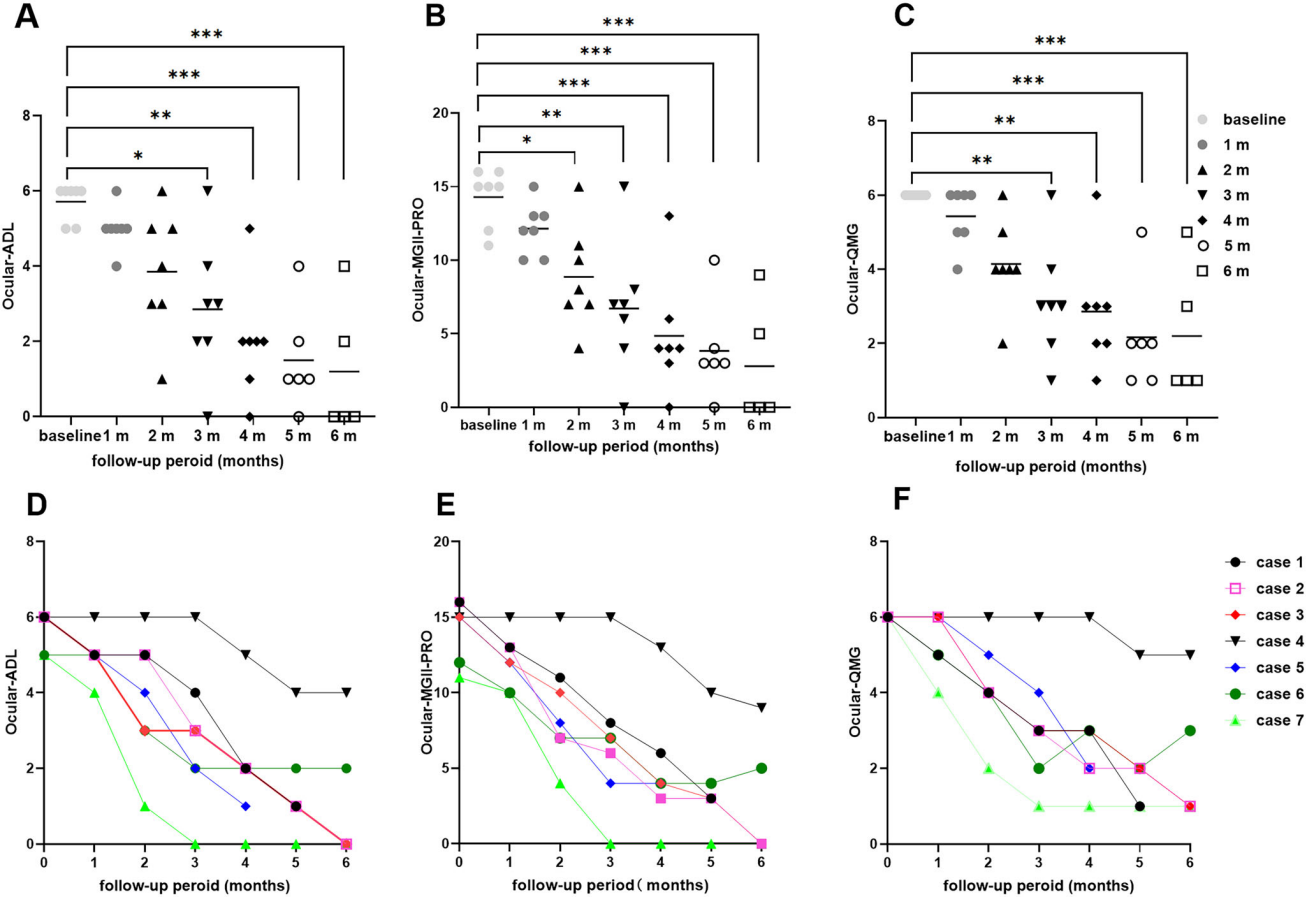
Clinical outcomes following telitacicept treatment in myasthenia gravis with refractory ocular symptoms: (A) and (C) There was a significant decline in median ocular ADL and QMG scores at the 3‐month follow‐up (*p* < 0.05), which was sustained through the 6‐month visit (*p* < 0.05). (B) A reduction in median ocular MGII‐PRO scores was observed at the 2‐month follow‐up (*p* < 0.01), with sustained efficacy through month 6 (*p* < 0.01). (D‐E) The change in each patient's ocular ADL, MGII‐PRO, and QMG scores post‐telitacicept treatment. QMG, Quantitative Myasthenia Gravis; MGII‐PRO, Myasthenia Gravis Impairment Index‐patient‐reported outcome; ADL, activities of daily living scores.

All patients completed at least 4 months of follow‐up, six reached 5 months, and five completed 6 months. At the first follow‐up after initiating telitacicept treatment, none had achieved CMI on the MG‐PIS. By the second follow‐up, four (Cases 3, 5, 6, and 7) demonstrated CMI with a decrease in ADL ≥ 2 points, and Case 7 achieved MSE. At the third follow‐up, six of seven patients fulfilled CMI, and Case 7 retained MSE. Case 4 remained unchanged until month 4, when some improvement was first documented. Six of seven patients exhibited CMI at the fourth follow‐up, and by the fifth follow‐up, four of six patients had achieved MSE (Figures 3, 4, 4D).

Additionally, at the 3‐month follow‐up, the seven patients showed statistically significant improvements from baseline in mean ocular ADL (2.86 ± 1.86 vs baseline) and QMG scores (3.14 ± 1.57 vs baseline; both *P* < 0.01). This improvement was sustained through the sixth month, as evidenced by the fourth follow‐up (ADL 2.00 ± 1.53, QMG 2.86 ± 1.57), the fifth follow‐up (ADL 1.50 ± 1.38, QMG 2.17 ± 1.47), and the sixth follow‐up (ADL 1.20 ± 1.79, QMG 2.20 ± 1.79, *P* < 0.01). Notably, ocular MGII‐PRO scores demonstrated early improvement at month 2 (4.14 ± 1.22, *P* < 0.01) with sustained efficacy through month 6 (2.4 ± 1.91, *P* < 0.01) (Figure 4). During telitacicept maintenance therapy, the steroid dose in Case 3 was reduced from 15 to 10 mg, while in Case 6, steroid treatment was continued at 5 mg. Case 4 discontinued telitacicept after the sixth follow‐up due to lack of further improvement. Case 6 remained on basic immunotherapy (TAC 2 mg and CS 5 mg) due to financial constraints after achieving symptom relief with telitacicept. The dosage of the underlying immunosuppressant was not adjusted for all patients during the treatment and follow‐up period.

### 3.3 Safety Profiles

During treatment with telitacicept in seven patients, no infections or allergic reactions were reported during telitacicept treatment in these seven patients. Two patients reported slight swelling and pain at the local injection site that resolved spontaneously without treatment. No significant adverse effects, relapses, or acute exacerbations were observed.

## 4 Discussion

In this retrospective real‐world study, telitacicept demonstrated both efficacy and safety in seven refractory ocular MG patients. Early clinical response emerged by 2 months, with four patients achieving CMI and one attaining MSE. By 3 months, 6/7 patients sustained CMI improvements, accompanied by statistically significant enhancements in ocular ADL, MGII‐PRO, and QMG scores. The therapeutic benefits with a maintained safety profile persisted through extended follow‐up.

Patients with refractory MG face a significant disease burden, including a higher risk of acute exacerbations, myasthenic crises, and disability. The condition also contributes to increased unemployment, stigma, social isolation, emotional dysfunction, and sleep disturbances, all of which adversely affect the overall quality of life (Schneider‐Gold et al. 2019; Schroeter et al. 2021; Harris et al. 2020; Cortés‐Vicente et al. 2022; Nagane et al. 2017). Similar burdens are observed in patients with refractory OMG. Our study demonstrates the efficacy of telitacicept in various refractory ocular cases, including refractory OMG (in both pediatric and adult patients), gMG with residual ocular muscle symptoms, childhood‐onset MG, early‐onset MG, antibody‐negative and antibody‐positive patients, and those with different disease durations, suggesting that telitacicept is a promising treatment option for refractory ocular symptoms. Recent reports have described the therapeutic application of novel monoclonal antibodies, such as efgartigimod and eculizumab, in managing residual ocular muscle symptoms (Weidmayer and Gallagher 2023; He et al. 2025). In our study, two patients had previously received efgartigimod; one adult experienced treatment failure, while one pediatric patient was switched to telitacicept due to persistent ocular symptoms despite systemic improvement. However, the use of eculizumab in refractory OMG is limited by insurance coverage and cost issues. CD20 monoclonal antibodies have also been employed in refractory MG, but their use is off‐label and associated with concerns regarding infection and safety (Zhao et al. 2021).

Telitacicept was developed to treat B‐cell‐mediated autoimmune diseases by inhibiting the development and survival of plasma cells and mature B cells (Dhillon 2021; Xie et al. 2022). Beyond its approval for SLE in China, telitacicept has also demonstrated clinical efficacy and safety in other autoimmune diseases, including primary Sjögren's syndrome (Xu et al. 2024), IgA nephropathy (Lv et al. 2023; Zan et al. 2024), IgG4‐related diseases (Cai et al. 2023), and neurological autoimmune disorders such as NMOSD (Ding et al. 2022) and gMG (Yin et al. 2024; Lin et al. 2024). Recent gMG studies (Yin et al. 2024; Lin et al. 2024) reported significant efficacy, with reductions in QMG or ADL scores across all muscle groups, including ocular muscles, sustained from week 12 to 24, Our findings align with these reports. In our case series, marked efficacy was noted at the 2‐month follow‐up using the standard telitacicept dose, with further CMI by 3 months. Notably, a phase 2 trial (Yin et al. 2024) showed earliest symptom improvement at week 4 with 240 mg weekly subcutaneously of telitacicept, demonstrating faster and more pronounced symptom relief compared to the 160 mg. However, the dose‐response relationship remains unclear. Pharmacokinetic studies in the Chinese population indicated that both total and free telitacicept followed linear pharmacokinetics within the 160–240 mg range, with maximum concentration and area under the curve increasing proportionally with dose (Xie et al. 2022). However, in our previous cohort study of telitacicept for refractory MG (Lin et al. 2024), individualized protocols with telitacicept also helped improve patients' clinical symptoms.

Five childhood‐onset MG cases were included in our study, and three pediatric patients were still experiencing refractory ocular symptoms when telitacicept was initiated as an add‐on therapy. Treatment options for pediatric MG, including OMG, have always been fraught with challenges. Long‐term and high‐dose corticosteroid use may have serious adverse effects in patients with MG (Benatar et al. 2016; Yan et al. 2025), with growth and developmental issues particularly prominent in pediatric patients. In our case series, three pediatric patients experienced corticosteroid‐related side effects. IVIG and plasma exchange treatments are often difficult to implement widely in pediatric patients because of accessibility and medical insurance issues. Other approaches, such as tacrolimus administration and thymectomy, have shown efficacy in long‐term treatment explorations in pediatric patients (Bi et al. 2022; Zhang et al. 2022; Liu et al. 2017). However, pediatric cases that are refractory and resistant to conventional treatments present even greater challenges. The application of novel biologics in pediatric patients also encounters the issue of off‐label use. Recently, eculizumab has been approved by the Food and Drug Administration for pediatric gMG in patients≥6 years (Ekroll et al. 2011). Our study demonstrated that telitacicept is a potential therapeutic candidate for children with refractory OMG.

In our study, Case 4 exhibited a lower response rate compared with the other patients. Specifically, some symptom improvement was noted between the fourth and fifth months of follow‐up; however, no further improvement in ocular muscle symptoms was observed despite continued treatment. Telitacicept was discontinued after the six‐month follow‐up. The unique physiological characteristics of extraocular muscles, which distinguish them from skeletal muscles, may contribute to the heterogeneity of individual clinical responses (Al‐Haidar et al. 2018), including the immature development of synaptic folds, ultra‐high motor neuron firing frequency, high proportion of tonic fibers, and low expression levels of complement regulatory proteins (e.g., CD55/CD59). These structural differences make extraocular muscles more susceptible to complement‐mediated damage (Shuey 2022; Melson et al. 2020). Therefore, complement‐targeted therapy could represent a valid subsequent treatment strategy in Case 4. Additionally, a lower response rate due to the long‐term disease course, potentially causing irreversible postsynaptic membrane damage in the extraocular muscles, cannot be excluded. Extraocular muscle atrophy with fatty infiltration has been commonly observed in a Greek cohort of patients with MG who experienced treatment challenges and frequent recurrence of ocular involvement (Velonakis et al. 2021). Early intervention is critical for preventing structural degeneration in patients with refractory diseases.

This study utilized the traditional MGFA classification, MG‐PIS, MGII‐PRO, ADL, and QMG scores to evaluate changes in ocular muscle symptoms in patients. However, these conventional methods have certain limitations in detecting subtle changes and assessing treatment response. An artificial intelligence‐assisted diagnostic system developed on a digital platform integrates computer vision and machine learning to measure the palpebral fissure height (for ptosis assessment) and quantitatively evaluate the degree of limitation in extraocular movement in six directions (for diplopia assessment), demonstrating greater sensitivity, objectivity, and reproducibility (Garbey et al. 2023; Lesport et al. 2024; Garbey et al. 2024). Additionally, dynamic high‐resolution contrast‐enhanced orbital magnetic resonance imaging and diffusion tensor imaging can be used to assess changes in extraocular muscle blood flow and observe alterations in the structure of extraocular muscle fibers, providing predictive value for disease activity and treatment response (Velonakis et al. 2021; Keene et al. 2022). These assessment methods are expected to provide OMG patients with simpler and more precise approaches in the future.

This study has several limitations: (1) its retrospective design; (2) the small sample size, which primarily included young childhood‐onset and early‐onset patients, with few late‐onset cases; (3) patient evaluation was based solely on clinical scores, lacking relevant serological markers or imaging evidence to further explore the potential mechanism of action of telitacicept; (4) Case 2 had a short OMG disease course, so the possibility of conversion to GMG cannot be entirely ruled out; (5) the study duration was inadequate for evaluating long‐term efficacy and safety; and 6) comprehensive immunopharmacological analysis is precluded by the retrospective nature of clinical observations. Future large‐sample, long‐term, well‐designed clinical trials are needed to confirm the efficacy and safety of telitacicept in refractory cases.

## Conclusion

Telitacicept has exhibited efficacy and safety in pediatric and adult patients with MG experiencing refractory ocular symptoms, demonstrating potential as a viable therapy for patients with refractory ocular myasthenia.

## Author Contributions

**Jing Lin**: formal analysis, investigation, methodology, writing – original draft. **Yue Li**: formal analysis, investigation, resources, writing – original draft. **Mengcui Gui**: formal analysis, investigation, resources, writing – original draft. **Bitao Bu**: conceptualization, formal analysis, writing – review and editing. **Zhijun Li**: conceptualization, formal analysis, methodology, writing – review and editing

## Funding

This work was supported by the National Science Foundation of China (82371411), the Natural Science Foundation of Hubei Province (2020CFB744), and the Health Commission of Hubei Province Scientific Research Project (WJ2021M119).

## Ethics Statement

This study received ethical approval from the Clinical Investigation Committee of Tongji Hospital, Tongji Medical College, Huazhong University of Science and Technology (approval number: TJ‐IRB202406006) and complied with the Declaration of Helsinki.

## Conflicts of Interest

The authors declared no conflicts of interest.

## Data Availability

The datasets generated and/or analyzed in this study are available from the corresponding author upon reasonable request.
